# EVALUATING GERIATRIC WORKFORCE DEVELOPMENT NEEDS AMONG HOSPITALS USING NICHE BENCHMARKING DATA

**DOI:** 10.1093/geroni/igz038.3458

**Published:** 2019-11-08

**Authors:** Roy A Thompson, Loretta Matters, Kirsten Corazzini, Eleanor McConnell

**Affiliations:** 1 Duke University School of Nursing, Durham, North Carolina, United States; 2 Duke University, Durham, North Carolina, United States; 3 University of Maryland School of Nursing, Baltimore, Maryland, United States

## Abstract

The Nurses Improving Care for Health System Elders (NICHE) program aims to improve geriatric care competencies for improved care quality. A quantitative descriptive design utilizing secondary data analysis was done to evaluate geriatric workforce enhancement efforts in one acute healthcare system. Data were collected using the Geriatric Institutional Assessment Profile (GIAP) from 2008 and 2013. The GIAP measures perceived professional issues (disagreements among staff and families, limited access to geriatric services, vulnerability to legal action, intensity and burden of behavioral problems) on a Likert scale from best=0 to poor=10. Staff perception of the Geriatric Care Environment was scored by the GIAP as: age sensitive care delivery (0-40), institutional values (0-28), resource availability (0-32) and capacity for collaboration (0-12). Higher scores on the Geriatric Care Environment reflected improvements. Independent sample t-tests examined changes in baseline scores. Post-NICHE implementation, compared to peer hospitals by teaching status and bed size in 3 hospitals there were significantly (p<0.05) improved scores for: access to geriatric services (2.79-3.21), burden of behavioral problems (2.40-3.15), aging sensitivity care delivery (26.05-29.53), institutional values (18.85-19.59) and resource availability (19.51-19.97). Peer hospitals had significantly (p<0.05) better scores for: disagreements among staff about treatment of older adults (1.63-1.94) and capacity for collaboration (7.72-7.99). Findings indicate improvement in perceived professional issues and need for improvement in the geriatric care environment and care redesign to progress to becoming an Age-Friendly health system. This was an initial step in a health system to improve care quality through health workforce development.

